# Safety of NADES Extract of *Glycyrrhiza* Roots After Topical Application and Peroral Administration to Mice

**DOI:** 10.3390/molecules30244704

**Published:** 2025-12-09

**Authors:** Veronika A. Shikova, Olga N. Pozharitskaya, Elena V. Flisyuk, Dmitry Yu. Ivkin, Dmitrii N. Borovikov, Olga L. Balabanova, Alexander N. Shikov

**Affiliations:** 1 Department of Pharmaceutical Formulations, St. Petersburg State Chemical Pharmaceutical University, Prof. Popov Str., 14, 197376 Saint-Petersburg, Russia; veronika.shikova@spcpu.ru (V.A.S.); elena.flisyuk@pharminnotech.com (E.V.F.); 2 Murmansk Marine Biological Institute of the Russian Academy of Sciences (MMBI RAS), Vladimirskaya Str., 17, 183038 Murmansk, Russia; olgapozhar@mail.ru; 3 Center of Experimental Pharmacology, St. Petersburg State Chemical Pharmaceutical University, Prof. Popov Str., 14, 197376 Saint-Petersburg, Russia; dmitry.ivkin@pharminnotech.com; 4 Infochemistry Scientific Center, School of Life Sciences, ITMO University, Lomonosov Str., 9, 191002 Saint-Petersburg, Russia; dima010203@ya.ru; 5 Department of Pharmaceutical Chemistry, St. Petersburg State Chemical Pharmaceutical University, Prof. Popov Str., 14, 197376 Saint-Petersburg, Russia; o.l.ya@mail.ru

**Keywords:** *Glycyrrhiza glabra*, NADES extract, toxicity, peroral administration, dermal toxicity, LC-MS/MS

## Abstract

Natural deep eutectic solvents (NADES) have been extensively used for the extraction of a wide spectrum of plant materials. However, limited data about the in vivo toxicity of NADES extracts restrict their future practical application. In this study, we are aiming to assess the safety of a Sorbitol–lactic acid (3:1 mol./mol.; 30% water) NADES extract of *Glycyrrhiza* roots (GR) in mice. LC-MS/MS analysis revealed the presence of 17 metabolites, including phenolic acids, flavonoids, their glycosides, chalcones, terpene saponins, and coumarins. Interestingly, most of the identified compounds were found in higher amounts in NADES extract compared to water and EtOH extracts. No skin edema, inflammation, or erythema was observed in mice after topical application of NADES extract of GR and NADES at the doses of 50, 100, and 150 µL/mice in comparison with the control group. The calculated primary irritation index was about 0.45 both for NADES and NADES extract of GR only in high doses and falls into mild irritant categories. The individual Draize scores indicate that erythema was evident in the first three days and that all signs had disappeared by day five. No acute toxic signs or mortality of animals was observed in mice following oral administration of single doses of 4, 6, and 20 g/kg of NADES or NADES extract of GR. The NADES and extract seem to be safe at doses of up to 20 g/kg, and the LD50 was considered to be >20 g/kg. Our results open prospects for the use of NADES extract of GR for the development of transdermal and peroral formulations in the cosmetic, food, and pharmaceutical industries.

## 1. Introduction

In 2013, Choi et al. (2011) promoted natural deep eutectic solvents (NADES) as a third category of liquids that are present in significant quantities in plant cells [1]. Since then, intensive research has begun on the use of NADES for extracting biologically active compounds from various plant sources [2,3,4,5,6]. The majority of scientists asserted the safety of NADES based on the safety of the components of solvents, which are primarily represented by weak organic acids, quaternary ammonium salts, amino acids, and sugars [7,8,9,10]. NADES components are derived from biological sources and generally regarded as safe; however, it should be acknowledged that a natural product is not automatically devoid of toxicity [11].

According to several in silico studies, sugar- and straight-chain alcohol-based deep eutectic solvents are classified as less toxic than organic acid- and quaternary ammonium-based solvents [11,12,13]. In vitro experiments on cytotoxicity were conducted on several microorganisms to evaluate the antimicrobial activity of NADES [14]. NADES containing organic acids demonstrated the highest inhibition of bacterial growth, aligning with in silico research findings. Furthermore, this suppression was more substantial than the impact of the acids on their own, which could be attributed to the synergistic interactions present in the NADES. De Morais et al. (2015), when assessing the toxicity of choline chloride (ChCl) and various organic acids on the bacterium *Vibrio fischeri*, noted the contrary trend, with the acid alone exerting a more significant effect than the NADES [15]. All the analyzed NADES displayed moderate toxicity when compared to the organic acids individually, with toxicity rising alongside acid concentration. In one of our recent studies, lactic acid-based NADES showed antimicrobial activity against gram-positive and gram-negative microorganisms and was associated with the water content and pH [16]. We believe that the above mentioned factors should be considered as benefits of NADES rather than as limitations for use because of toxicity.

The cytotoxic effects of NADES were shown in multiple cell lines. Paiva et al. (2014) described the significant cytotoxic impacts of NADES containing tartaric and citric acids on L929 fibroblast-like cells [17]. The results of cytotoxicity experiments on human cancer cell lines (HeLa, MCF-7, and HEK293T) support the data on high antimicrobial activity of organic acid-based NADES and confirm the significant impact of pH on toxicity [14]. The effect of ChCl-based NADES was studied on cancer cell lines (HT-29, Caco-2, and MCF-7) and normal fibroblast lines (MRC-5). Combinations of ChCl with citric and malic acids were found to be the most cytotoxic, especially with respect to the HT-29 cell line, while the antiproliferative activity of NADES was low against normal fibroblast MRC-5. The inhibition correlated with the sample’s pH, since more acidic mixtures (pH 1.5) exhibited greater cytotoxicity [18]. Another consequence of NADES that could be significant for cytotoxicity is the modification of osmotic equilibrium. The presence of NADES may disrupt the osmotic pressure of the environment, leading to a heightened cytotoxic impact, as demonstrated in MCF-7 cells by McGrail et al. [19].

In vivo, the LD_50_ for ChCl:glycerol DES (1:3) in ICR mice was 6.39 ± 0.53 g/kg, while the LD_50_ of DESs was less than their individual components [20]. When the toxicity of ChCl:glycerol (1:2) NADES was tested on mice after oral administration, the NADES was considered safe with an LD_50_ estimated as 7733 mg/kg [21]. In a two-week toxicity study, Wistar rats received peroral 3 mL of water or 3 mL of NADES (betaine–glycerol 1:2 with 10% water) extract from green coffee beans twice daily. Two out of six rats died following treatment with the NADES extract. NADES treatment led to increased water intake, decreased food consumption and weight loss, liver enlargement, and plasma oxidative stress linked to elevated blood lipid levels. The pure NADES was not evaluated [22]. The pretreatment of rats with ethanol-induced gastric ulcers using ChCl–glycerol–citric acid NADES (0.5:2:0.5) and NADES-based blueberry extract for 14 days exhibited significant protection against both macroscopic and histopathological gastric injuries caused by EtOH, decreased glutathione depletion, protein oxidation, nitric oxide overproduction, and the inflammatory response [23]. Objectively, animal studies on the toxicity of NADES are limited and indicate a significant influence of solvent components and the nature of the plant matrix.

*Glycyrrhiza* spp. attract the attention of scientists due to the wide pharmacological activities associated with various classes of bioactive metabolites. With the increasing popularity of green chemistry and green solvents, *Glycyrrhiza* spp. have been extracted using NADES. Being an important metabolite, Glycyrrhizic acid (GA) is considered a target component of licorice and used for assessment of the efficiency of extraction of licorice roots by NADES. Dynamic maceration with stirring using NADES (ChCl–lactic acid; 1:1; 30% water) was found as an optimal condition for the extraction of *Glycyrrhiza glabra* L., leading to a NADES extract with 53.72 ± 0.57 mg/g of GA yield [24]. In a further study, the same group of authors, using the same NADES but after ultrasonic extraction, obtained a NADES extract with a GA concentration of 55.11 mg/g. The extraction time was reduced to 15 min [25]. NADES were demonstrated as suitable solvents for extraction of flavonoids from tablets containing *G. uralensis* extract. Among eighteen tested binary or ternary combinations, NADES comprising 1,4-butanediol–levulinic acid (1:2; 17% water) was optimal for extraction of five components, namely liquiritin, isoliquiritin, liquiritigenin, GA, and isoliquiritigenin, using ultrasound-assisted extraction [26]. Nevertheless, we have not found in the available literature results of more detailed analysis of NADES extracts from *Glycyrrhiza* spp. Information about in vivo safety of licorice NADES extracts is not available.

While certain NADES have been noted to exhibit toxicity, it is still less than that of conventional volatile organic solvents. For NADES to gain broad acceptance in the food, cosmetic, and pharmaceutical sectors it is crucial to collect more extensive information on their toxicity, especially in vivo. In this study, we are aiming to assess the safety of a NADES extract of *Glycyrrhiza* roots after topical application and peroral administration to mice.

## 2. Results and Discussion

### 2.1. Analysis of Metabolites in Extracts

**Figure 1:**
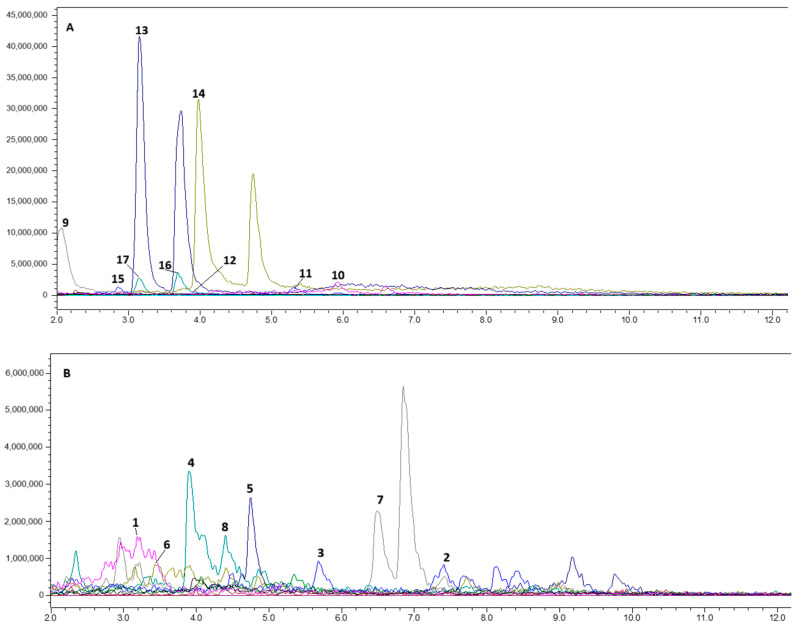
Typical total ionic chromatogram of NADES extract from *Glycyrrhiza* roots: (**A**)—negative ionization; (**B**)—positive ionization.

Water, EtOH, and NADES extracts of *Glycyrrhiza* roots were analyzed by LC-MS/MS. The typical chromatograms for NADES extract in both negative and positive modes are presented in Figure 1, while the list of metabolites annotated in water, EtOH, and NADES extracts of *Glycyrrhiza* roots is presented in Table 1.

The metabolite profile of *Glycyrrhiza* roots in our study is in agreement with previous publications [28,29,30,33]. To our knowledge, the ability of water, ethanol, and NADES to extract metabolites from licorice roots has not yet been compared in the scientific literature. Previously, liquiritin, isoliquiritin, liquiritigenin, GA, and isoliquiritigenin were extracted by NADES from tablets with licorice extract [26]. In the current study, we have found 17 active metabolites from *Glycyrrhiza* roots using sorbitol and lactic acid-based NADES. In positive ionization mode, we annotated some phenolic acids, flavonoids, and chalcones. The literature indicates that ferulic acid (**1**), a nearly non-toxic phenolic acid, reduces cholesterol levels and protects against coronary artery disease. It also has antibacterial, anti-inflammatory, antithrombotic, and anticancer effects [37,38]. In addition to ferulic acid (**1**), other hydroxy-phenolic acids, sinapic (**7**) and p-coumaric (**15**) acids, were annotated in the positive and the negative ionization modes, respectively. A wide range of pharmacological activities have been described in the literature for hydroxycinnamic acids, including antioxidant, anticancer, antitumor, antidiabetic, anti-inflammatory, antimicrobial, anticholesterolemic, antimutagenic, and antihypertensive [39]. We have not detected (**7**) in the water and EtOH extracts, but it was extracted by NADES. Ferulic acid (**1**) was annotated in all extracts, but its content in the NADES extract was 20 times higher than in the alcoholic one. Compound (**15**) was approximately 100 times more efficiently extracted by NADES than by water and was not detected in the EtOH extract.

Flavonoids, glyasperin C (**2**), and kaempferol 7-*O*-glycoside (**5**), contribute to anti-inflammatory, antimicrobial, wound healing, and other activities [40,41,42]. The above mentioned compounds were annotated in all samples. After peak integration, we found that gliasperin C was extracted by NADES 8–10 times more efficiently than by water and EtOH. The content of kaempferol 7-*O*-glycoside in the NADES extract was 87 times higher than in the water extract.

Chalcones such as isoliquiritin (**4**), licochalcone B (**8**), and naringenin (**6**) are able to significantly reduce oxidative stress and inhibit free radical production by the body [43,44]. Some authors noted these compounds as neuroprotective agents [44]. Notably, (**4**) and (**8**) have been extracted only from licorice [45]. Glycyuralin B (**10**) also belongs to this class of chalcones. Glycyuralin is able to inhibit PTP1B and α-glucosidase, regulating blood glucose levels [46]. In these experiments, (**4**) was extracted by NADES 8 times better than by the selected protonic solvents. Compound (**6**) was extracted 24 and 14 times more efficiently than by water and ETOH, respectively. Licochalcone B (**8**) was found in the NADES extract at 87 times the level in water and 14 times the level in EtOH. The content of (**10**) in the NADES extract was equal to that in the aqueous extract and 28 times higher than the content in the EtOH extract.

Antiatherogenic, anticancer, antidiabetic, antimicrobial, anti-inflammatory, antiviral, anti-asthmatic, hepatoprotective, and melanin-inhibitory effects have been documented for the glycyrrhizic acid (**3**), isoliquiritin (**4**), and glucuronic acid (**9**) [45,47,48,49,50]. Some studies associate UV-B protective effects with glycyrrhizic acid [51]. Glycyrrhizic acid is effective for the treatment of atopic dermatitis and can increase skin penetration for some drugs [52,53]. Lee et al. (2013) have demonstrated antispasmodic effects for glycyrrhizic acid (**3**), isoliquiritin (**4**), and liquiritigenin (**13**) [54]. In water and EtOH extracts we have detected fragments of disaccharides (*m*/*z* 351), and aglycone—glycyrrhetinic acid (*m*/*z* 471), indicating hydrolysis of glycyrrhizic acid. However, no glycyrrhizic acid fragments were found in the NADES extract. In a comparison of the integral peak areas of glycyrrhizic acid in NADES, aqueous and ethanol extracts showed a 2-fold higher content in NADES compared to the aqueous extract with an equal content in the EtOH extract.

Inflacoumarin A (**11**), annotated in negative ionization mode in all studied extracts in equal amounts, is mentioned in the literature as an antimicrobial agent against methicillin-resistant *Staphylococcus aureus* [55]. In the negative ionization mode, we annotated flavonoids: isoviolanthin (**12**), liquiritin (**14**), liquiritigenin, (**13**) rutin (**17**), and flavonoid glycoside quercetin 3-*O*-glycoside (**16**). As reported by Wang et al. (2023), due to its antioxidant properties, (**12**) can act as a protective agent for the skin, probably increasing the viability of skin keratocytes [56]. The content of (**12**) in the aqueous and EtOH extracts was 10 times higher than the content in the NADES extract. Compound (**14**) exhibits various pharmacological properties such as anti-Alzheimer’s activity, antidepressant effects, antitumor capabilities, anti-inflammatory responses, cardiovascular protection, antitussive action, liver protection, and skin safeguarding effects [57]. Liquiritigenin (**13**), an aglycone of (**14**), can contribute to the estrogen-like and anti-inflammatory activity, exerting an agonistic effect on estrogen receptors-β, which is important in the treatment of age-related diseases, cancer, and diabetes [58,59,60,61,62]. Liquiritin and liquiritigenin were detected in all studied samples. The levels of these compounds in the NADES extract were 20- and 40-fold higher than those in the aqueous and ethanol extracts, respectively. Notably, (**17**) and its partial hydrolysis product (**16**) exhibit antimicrobial activity including antibiotic-resistant *St. aureus* and *Escherichia coli* [63]. All extracts analyzed by us contained (**16** and **17**). NADES extract was enriched with (**16**) by 5-fold compared to the water extract and by 61-fold compared to the EtOH extract. The content of (**17**) in the NADES extract was 12-fold higher than in EtOH extract and 19-fold higher than in the water extract.

### 2.2. In Vivo Study of the Safety of NADES Extract of Glycyrrhiza Roots

Our results of the chemical characterization of extracts evidenced about the wide specter of active metabolites extracted from *Glycyrrhiza* roots (GR) and the high efficiency of NADES as an ecxtragent. The literature data supports the high therapeutic potential of NADES extract for topical or oral applications. However, limited data about the in vivo dermal or peroral toxicity of NADES extracts restrict its future practical application.

#### 2.2.1. Skin Irritation Assessment

**Figure 2:**
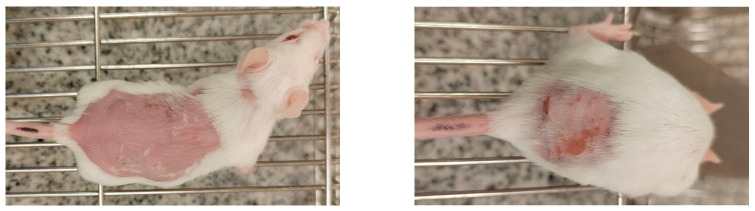
Representative photographs of the lowest (0) and highest (4) possible erythema scores in mice. All photos are orientated with the head of the animal to the right and the tail to the left.

**Figure 3:**
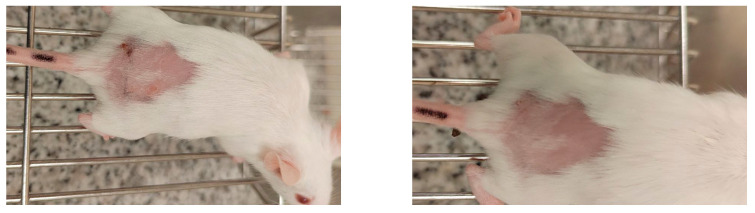
Photograph of mice on days 3 and 5 after application of 150 μL of NADES extract. All photos are orientated with the head of the animal to the right and the tail to the left.

Table 2 showed skin tolerance test results in mice. No skin edema was observed in mice after application of NADES extract of GR and pure NADES in comparison with the control group. The treated skin was intact; no inflammation or erythema compared to the untreated site (Figure 2). Calculated primary irritation index (PII) recorded in tested samples was about 0.45 both for pure NADES and NADES extract of GR only in high doses. For the control group, this index was 0.06. Thus, all of them fall into the mild (PII < 2) irritant category. The individual Draize scores indicate that the erythema was evident in the first 3 days and that all signs had disappeared by day 5 (Table 2) (Figure 3).

No skin ulcerations or skin edema were noted in all groups after application of 50 or 100 µL/mice of tested substances. Thus, both pure NADES and NADES licorice root extract can be classified as non-irritating substances. Recent reviews have described the toxic effects of licorice and glycyrrhizin after several routes of administration [64,65]. To our knowledge, this is the first study on the toxicity of NADES and NADES licorice extract after topical application.

#### 2.2.2. Acute Dermal Toxicity

Clinical assessments of the laboratory mice in all experimental groups over five days indicated that their overall health was satisfactory. No behavioral changes were noted, no seizures were observed, and appetite and thirst were maintained. Response to tactile, pain, sound, and light stimuli was adequate. Skin integrity was intact, elasticity was preserved, and hyperemia was absent. The color of visible mucous membranes was normal. Respiratory rate and depth, as well as heart rate, were unchanged. Feces were dark brown, firm, and of a characteristic oval-oblong shape with a specific odor. The amount of feces corresponded to the volume of feed consumed.

Observation indicated that none of the animals were found either in a moribund condition nor showing any severe pain and/or enduring signs of severe distress [66]. There were no animal mortality rates in any experimental group during the study. Furthermore, animals in all groups gained weight uniformly throughout the experiment (Table 3).

The data were normally distributed. A repeated-measures ANOVA revealed no effect of the “Group” factor on mouse body weight dynamics on the 3rd (F = 1.46; *p* = 0.24) and 5th (F = 1.55; *p* = 0.22) days of observation, nor did the “Dose” factor influence body weight dynamics on the 3rd (F = 2.04; *p* = 0.14) and 5th (F = 0.75; *p* = 0.48) days. No significant difference was found in the percentage of body weight gain between mice in the NADES and NADES extract of GR groups compared to control animals.

Additionally, testing of pure NADES used for GR extraction revealed that the solvent also had no topical toxicity. An assessment of body weight dynamics and observation of the general condition of the animals showed that a single topical application of NADES to experimental animals did not affect behavioral reactions; there was no clinical picture of intoxication throughout the experiment; all animals were in satisfactory condition. We have not found data from the literature about the toxicity of NADES (or NADES extracts) after topical application to mice. Nevertheless, the non-volatility of the majority of NADES is beneficial from a toxicity perspective, as it results in reduced toxicity, particularly for topical use.

#### 2.2.3. Acute Peroral Toxicity

The acute toxic effect of NADES and NADES extract of GR was determined with the limit test dose of 20 g/kg (Table 4). No treatment-related toxic symptoms or mortality were observed after oral administration of the tested samples of 4, 6, and 20 g/kg. The general behavior of the extract-treated animals and the control group was observed, first for a short period (4 h) followed by a long period (72 h), and did not display any drug-related changes in behavior, breathing, skin effects, water consumption, impairment in food intake, and temperature. No animal mortality was observed in the next 4–14 days. Therefore, the extract seems to be safe at a dose level of 20 g/kg, and the LD_50_ was considered to be >20 g/kg.

The data were normally distributed. A repeated-measures ANOVA revealed no effect of the “Group” factor on mouse body weight dynamics on the 2nd (F = 0.85; *p* = 0.37) and 3rd (F = 0.79; *p* = 0.39) days of observation, nor did the “Dose” factor influence body weight dynamics on the 2nd (F = 0.64; *p* = 0.54) and 3rd (F = 0.48; *p* = 0.63) days. No significant difference was found in the percentage of body weight gain between mice in NADES and NADES extract of GR experimental groups compared to control animals (Table 4). An assessment of body weight dynamics and observation of the general condition of the animals showed that a single oral administration of the test substance to experimental animals did not affect behavioral reactions; there was no clinical picture of intoxication during the experiment; all animals were in satisfactory condition.

Some authors have observed different toxicity of NADES and NADES extract of phenolics. In particular, the NADES (ChCl–glycerol) extract from *Sideritis scardica* exhibited a stronger antiproliferative effect on CCL-1 cells (mouse fibroblasts) than the cytotoxicity of pure NADES [67]. In another study, the same NADES was more cytotoxic against MCF-7 cells in comparison with sunflower meal phenolic NADES extract, while when using urea–glycerol NADES, the antiproliferative activity of sunflower meal NADES extract was superior to that of the corresponding NADES [68]. NADES extract of GR is rich in phenolic compounds (Table 1). However, we did not observe oral toxicity for either NADES or NADES extract of GR at doses up to 20 g/kg. Therefore, we are unable to assess the impact of extracted compounds on toxicity. The data on the toxicity of EtOH and aqueous extracts of *G. glabra* were summarized in review [64]. No mortality of rats was observed at >1000 mg/kg and in mice at doses of >1500 mg/kg, respectively. When we tested NADES extract of GR, no mortality of mice was observed at doses up to 20 g/kg. Popović et al. (2023) demonstrated that the inhibition of HT-29 cell line growth correlated with pH of NADES, since more acidic NADES (pH 1.5) exhibited greater cytotoxicity [18]. Although the pH of the NADES tested by us was about 1.5 [16], no toxicity after peroral administration was found in mice. The pH of the NADES extract of GR increased up to 3.3 due to extracted active metabolites of GR. Nevertheless, we have not found toxicity of NADES extract.

The literature data on the oral toxicity of NADES are very rare. In an acute toxicity study of NADES choline chloride–glycerol (ChCl-GL, molar ratio 1:2), it was found that with an increase in dose to 6160 mg/kg and higher, most mice showed excitement, jumped, and then within 20–30 s decreased activity, dyspnea, convulsions, tremors, and other symptoms appeared. Thirty of the seventy mice died within 4 h, and the survivors returned to normal within 2 h. Moreover, mortality increased with an increase in the oral dose of ChCl-GL (1:2). No significant effect on body weight gain was observed between the control and experimental groups during the observation period. According to the Karber method, the calculated LD_50_ was 7733 mg/kg with a 95% confidence interval of 7130–8387 mg/kg when administered orally. This indicates that ChCl-GL (1:2) is non-toxic and relatively safe for clinical use in oral dosage forms [21]. Hayyan et al. tested single doses of ChCl:GL (1:3 mole ratio) NADES of 5000, 10,000, and 20,000 mg/kg of body weight on mice and reported an LD_50_ of 6400 mg/kg [20]. In the current study, the single dose of NADES (Sorbitol–lactic acid; 3:1), as well as of NADES extract of GR, was 20,000 mg/kg of initial mice body weight, and no mortality was observed. We can attribute this fact to the composition of NADES.

## 3. Materials and Methods

### 3.1. Materials and Reagents

The roots of *G glabra* L. were provided by Krasnogorskleksredstva JSC (Krasnogorsk, Russia). *D*-sorbitol (>98.0%) was purchased from Sigma-Aldrich, *L*-lactic acid (88.0–92.0%) was from Panreac Química SLU (Barcelona, Spain). Water was purified by Milli-Q system (Millipore, Bedford, MA, USA). Other reagents of HPLC grade were obtained from local suppliers.

### 3.2. NADES Preparation and Extraction of Plant Material

The NADES was prepared by the heating method [7]. Sorbitol and lactic acid (3:1 M/M) were mixed and heated to 70 °C for 60 min with agitation at 400 rpm, and 30% of water (*v*/*v*) was added to NADES until a clear liquid was formed. Components of NADES and water content were selected based on our previous studies [69]. Extraction (1 g of roots with 25 mL of NADES, or water, or 50% EtOH) was performed with stirring (400 rpm) at 40 °C for 30 min.

### 3.3. Analysis of Metabolites

The analysis of plant metabolites was performed by Liquid Chromatography-Mass Spectrometry (LC-MS/MS) with triple quadrupole LCMS-8050 including vacuum degasser DGU-205R, system controller CBM-20A Lite, high-pressure pumps LC-20ADxr (Nexera XR), autosampler SIL-20ACxr, thermostat CTO-20AC, and changeover valve FCV-20AH2 (Shimadzu Corp., Kyoto, Japan). An analytical column Kinetex XB-C18 (Phenomenex, Torrance, CA, USA) was employed for the analysis. Data processing was carried out using LabSolutions LCMS software Version 5.97 SP1 (Shimadzu Corp., Kyoto, Japan). Mobile phase included solvent A: 0.63 g ammonium formate +1 mL of formic acid + deionized water to 1 L, and Solvent B: CH_3_CN. The gradient elution was as follows: 0–10 min, 5–90% B, followed by 2 min isocratic elution. Mobile phase flow rate: 0.32 mL/min. Column oven: 45 °C. Injection volume: 10 µL.

Briefly, 100 µL of EtOH extract was dissolved in 900 µL of mobile phase. For NADES extract, re-extraction was performed in butyl acetate. After that, the dry residue was dissolved in 1 mL of mobile phase. Mass spectrometric analysis was performed in the electrospray ionization (ESI) full scan mode. Collision energies (CE) were +22 eV and −22 eV, for positive and negative ionization modes, respectively. All samples were analyzed in triplicate. Online database http://www.massbank.jp (accessed on 5 November 2025) and the literature data were used for annotation of metabolites in extracts.

### 3.4. Animals Experiments

Female outbred mice (5–6 weeks old, *n* = 96) were obtained from Rapplovo animal house (Leningrad region, Russia). Only females were purchased for this study to reduce the risk of skin injury due to fighting, which is more common among male mice [70]. Upon arrival, the animals were acclimatized for a minimum of 10 days. All animals were randomly assigned to treatment groups, weighed, and individually identified via tail marking using a permanent marker. Dose groups were identified by cage cards. Both the dosing group as well as the animal numbers were identified on each cage. The animals were kept under standard conditions with a 12 h light–dark cycle, at an ambient temperature (22 ± 2 °C), and relative humidity of 60 ± 10%. They had free access to food (Standard diet: Volosovo, Russia) and water ad libitum. The mice were handled in accordance with European Union legislation [71] and the Russian Manual for laboratory animals [72]. All experiments were approved by the Bioethical Commission of the Federal State Budgetary Educational Institution of Higher Education SPCPU of the Russian Ministry of Health (ethical approval protocol #Mice/NADES-2024).

Before an experiment to test toxicity after topical application, mice were randomly assigned to nine groups (*n* = 6). The dorsal hair of the mice in an area of 2 × 2 cm was gently removed by shaving. The animals were immobilized during the procedure with light anesthesia using isoflurane. NADES and NADES extract of GR were topically applied to the denuded area of mice on the day after hair removal with single doses of 50, 100, and 150 µL. The animals in the control groups were applied saline at the same doses (Table 2 and Table 3). After the topical application, the animals remained in isolated boxes for 30 min to allow time for the drug’s absorption. Each mouse was carefully observed for the first 4 h, periodically for the first 24 h, and once daily for 5 days. During this period, animal activity related to motor-muscle coordination was analyzed. The body weight of animals was recorded on first, third, and fifth days (Table 3).

Skin tolerance tests were performed using the Organization for Economic Cooperation and Development guidelines [73] with a slight modification. Hairless areas were photographed immediately after shaving and every 24 h thereafter. Three reviewers who were blind to the treatment group used a modified Draize Skin Test scoring system [74] to assess the photographs (Figure 1). Each reviewer scored the treated skin for erythema (score 0 to 4). In addition, the skin was scored for edema (Figure 1) immediately after shaving and once daily by the same researcher. The edema score was recorded without the use of photographs because the three-dimensional nature of edema was not visible in photographs, as was determined during data collection for the pilot study. The researcher who recorded the edema score was not blind to the treatments. The test sites were then critically examined for dermal reaction using Draize scoring criteria, and the Primary Irritation Indexes (PII) of test substances were calculated [73,75]. The tested samples were then classified according to the Draize method of classification using the PII scoring as mildly irritant if (PII < 2), moderately irritant if (2 ≤ PII ≤ 5), and severe irritant if (PII > 5).

The acute toxicity after peroral administration was tested in accordance with [72]. Mice were randomized and assigned to seven groups (*n* = 6 per group) and fasted overnight before the experiment. Animals were administered by gavage with a single dose of NADES or NADES Extract of GR at the doses of 4, 6, and 20 g/kg, respectively. NADES and NADES Extract of GR were administered without dilution. The control group received 6 g/kg of saline. Each mouse was carefully observed for the first 4 h, periodically for the first 24 h, and once daily for 3 days. During this period, animals’ activity related to motor-muscle coordination was analyzed. The body weight of animals was recorded on the 1st, 2nd, and 3rd days (Table 4). Animals were monitored for the next 4–14 days for mortality. Since no animal deaths were observed in any of the experimental groups when the test substances were administered orally at doses up to 20 g/kg, it was not possible to calculate the LD_50_. Both NADES and the NADES extract of GH can be considered safe.

### 3.5. Statistical Analysis

Weight and Draize skin scores are shown as means ± SEM. Draize skin scores are categorical data and, hence, cannot have normal distributions. Therefore, significance was tested for those measures by using the nonparametric Wilcoxon method. Weight was analyzed using repeated-measures ANOVA. Data were statistically analyzed using STATGRAPHICS Centurion XV (StatPoint Technologies Inc., Warrenton, VA, USA), and *p* ≤ 0.05 was used to determine statistical significance.

## 4. Conclusions

In this study, we have evaluated for the first time the safety of NADES extract of *G. glabra* roots after topical application and peroral administration to mice. LC-MS analysis revealed the presence of 17 metabolites, including phenolic acids, flavonoids, their glycosides, chalcones, terpene saponins, and coumarins. Interestingly, a majority of the identified compounds were found in higher amounts in NADES extract compared to water and EtOH. The literature data on the pharmacological activity of found metabolites indicate that NADES extract of GR could be used for the development of formulations for topical and peroral administration.

In a skin irritation test, no edema, inflammation, or erythema was observed in mice after topical application of NADES extract of GR and NADES at the doses of 50, 100, and 150 µL/mice in comparison with the control group, which was treated with saline. The calculated primary irritation index was about 0.45 for both NADES and NADES extract of GR only in high doses and falls into the mild irritant category. The individual Draize scores indicate that erythema was evident in the first three days and that all signs had disappeared by day five. No acute toxic signs or mortality of animals were observed in mice following oral administration of single doses of 4, 6, and 20 g/kg of NADES or NADES extract of GR. The NADES and extract seem to be safe at doses up to 20 g/kg, and the LD_50_ was considered to be >20 g/kg.

In our current work, we have examined the safety of NADES and NADES extract of GR in one species of female animals. Future, more detailed safety studies, including sub-chronic toxicity and bio-chemical characterization of tissues and organs of animals, would be of interest.

The results of our pioneering study have proven the safety of NADES and NADES-GR extract and provide a basis for the development of innovative formulations with NADES extract of GR for the food, cosmetics, and pharmaceutical industries.

## Figures and Tables

**Table 1:** Metabolites annotated in water, EtOH, and NADES extracts of *Glycyrrhiza* roots.

No	Assignment	*m*/*z* Observed	Elemental Formula	Extract *	Ref
1	Ferulic acid	159 [M + H]+	C_10_H_10_O_4_	W, E, N	[27]
2	Glyasperin C	330 [M + H]+	C_21_H_24_O_5_	W, E, N	[28]
3	Glycyrrhizic acid	826 [M + H]+	C_42_H_62_O_16_	W, E, N	[29,30]
4	Isoliquiritin	429 [M + H]+	C_21_H_22_O_9_	W, E, N	[31]
5	Kaempferol 7-*O*-Glycoside	499 [M + H]+	C_22_H_27_O_13_	W, E, N	[32]
6	Naringenin	273 [M + H]+	C_15_H_12_O_5_	W, E, N	[32]
7	Sinapic acid	225 [M + H]+	C_11_H_12_O_5_	N	[33]
8	Licochalcone B	287 [M + H]+	C_16_H_14_O_5_	W, E, N	[28]
9	Glucuronic acid	193 [M−H]−	C_6_H_10_O_7_	W, N	[33]
10	Glycyuralin B	353 [M−H]−	C_20_H_19_NO_5_	W, E, N	[28,34]
11	Inflacoumarin A	321 [M−H]−	C_20_H_18_O_4_	W, E, N	[29]
12	Isoviolanthin	577 [M−H]−	C_27_H_30_O_14_	W, E, N	[29]
13	Liquiritigenin	417 [M−H]−	C_21_H_22_O_9_	W, E, N	[28]
14	Liquiritin	255 [M−H]−	C_15_H_12_O_4_	W, E, N	[29]
15	*p*-Coumaric acid	163 [M−H]−	C_9_H_8_O_3_	W, N	[35]
16	Quercetin 3-O-Glycoside	463 [M−H]−	C_21_H_20_O_12_	W, E, N	[36]
17	Rutin	609 [M−H]−	C_27_H_30_O_16_	W, E, N	[36]

* Water extract (W); EtOH extract (E); NADES extract (N).

**Table 2:** Average irritation scores after application of NADES and NADES extract of *Glycyrrhiza* roots in mice.

Group	Dose, µL/Mice	Draize Scores					
		1 h		3rd Day		5th Day	
		Erythema	Edema	Erythema	Edema	Erythema	Edema
Control	50/100/150	0/0/0	0/0/0	0/0/0.33	0/0/0	0/0/0	0/0/0
NADES	50/100/150	0/0/0	0/0/0	0/0/2.83	0/0/0	0/0/0	0/0/0
NADES extract of GR	50/100/150	0/0/0	0/0/0	0/0/2.67	0/0/0	0/0/0	0/0/0

**Table 3:** The effect of a single dose topical application of the tested substances on the body weight of mice, g (M ± SEM, *n* = 6).

Group	Dose, µL/Mice	Dynamic of Body Weight, g		
		1st Day	3rd Day	5th Day
Control	50 100 150	25.72 ± 0.77 27.05 ± 0.03 26.02 ± 0.58	26.44 ± 0.93 27.82 ± 0.93 27.07 ± 0.62	27.08 ± 0.86 27.70 ± 0.86 27.34 ± 0.75
NADES	50 100 150	25.14 ± 0.73 26.11 ± 0.89 26.83 ± 0.92	25.59 ± 0.87 26.69 ± 0.93 26.59 ± 0.69	25.94 ± 0.89 27.18 ± 0.76 26.48 ± 0.76
NADES extract of GR	50 100 150	26.43 ± 0.71 27.45 ± 0.58 26.51 ± 0.53	26.83 ± 0.69 28.04 ± 0.49 26.95 ± 0.53	27.16 ± 0.47 27.38 ± 0.46 27.68 ± 0.40

**Table 4:** The effect of a single peroral administration of NADES and the NADES extract of GR on the body weight of mice, g (M ± SEM, *n* = 6).

Group	Dose, g/kg	Dynamic of Body Weight, g		
		1st Day	2nd Day	3rd Day
Control	6	25.92 ± 1.01	26.03 ± 1.03	26.32 ± 1.00
NADES	4	25.61 ± 0.44	25.70 ± 0.37	25.98 ± 0.32
	6	25.69 ± 0.27	25.82 ± 0.05	25.95 ± 0.11
	20	24.77 ± 0.25	25.30 ± 0.37	25.32 ± 0.29
NADES extract of GR	4	25.27 ± 0.58	25.46 ± 0.40	25.61 ± 0.35
	6	25.74 ± 1.41	25.96 ± 0.99	26.11 ± 1.09
	20	28.03 ± 1.05	28.51 ± 0.90	28.57 ± 0.83

## Data Availability

The original contributions presented in this study are included in the article. Further inquiries can be directed to the corresponding author.
